# pH-driven enhancement of anti-tubercular drug loading on iron oxide nanoparticles for drug delivery in macrophages

**DOI:** 10.3762/bjnano.12.84

**Published:** 2021-10-07

**Authors:** Karishma Berta Cotta, Sarika Mehra, Rajdip Bandyopadhyaya

**Affiliations:** 1 Centre for Research in Nanotechnology and Science, IIT Bombay, Powai, Mumbai, Maharashtra – 400076, India; 2 Chemical Engineering Department, IIT Bombay, Powai, Mumbai, Maharashtra – 400076, India

**Keywords:** drug-nanoparticle interactions, drug uptake, intra-macrophage, iron oxide nanoparticles, norfloxacin

## Abstract

Nanoparticle deployment in drug delivery is contingent upon controlled drug loading and a desired release profile, with simultaneous biocompatibility and cellular targeting. Iron oxide nanoparticles (IONPs), being biocompatible, are used as drug carriers. However, to prevent aggregation of bare IONPs, they are coated with stabilizing agents. We hypothesize that, zwitterionic drugs like norfloxacin (NOR, a fluoroquinolone) can manifest dual functionality – nanoparticle stabilization and antibiotic activity, eliminating the need of a separate stabilizing agent. Since these drugs have different charges, depending on the surrounding pH, drug loading enhancement could be pH dependent. Hence, upon synthesizing IONPs, they were coated with NOR, either at pH 5 (predominantly as cationic, NOR^+^) or at pH 10 (predominantly as anionic, NOR^−^). We observed that, drug loading at pH 5 exceeded that at pH 10 by 4.7–5.7 times. Furthermore, only the former (pH 5 system) exhibited a desirable slower drug release profile, compared to the free drug. NOR-coated IONPs also enable a 22 times higher drug accumulation in macrophages, compared to identical extracellular concentrations of the free drug. Thus, lowering the drug coating pH to 5 imparts multiple benefits – improved IONP stability, enhanced drug coating, higher drug uptake in macrophages at reduced toxicity and slower drug release.

## Introduction

Nanoparticles have taken the center-stage in drug delivery applications, wherein they can improve drug pharmacokinetics and pharmacodynamics and may also increase drug accumulation in both animal cells and bacteria, proving beneficial to overcome drug resistance [1–2]. Iron oxide nanoparticles (IONPs), due to their biocompatibility and magnetic properties, have found applications in drug delivery, magnetic resonance imaging and treatment of iron deficiencies [3–6]. The property of hyperthermia has been found to be beneficial in localized drug release, particularly in cancer therapy [7]. In anti-cancer therapy, IONPs have also proven to be beneficial in overcoming multidrug resistance by enabling an increased drug uptake [8]. Similarly, physical combination of stabilized IONPs and anti-tuberculosis drugs improve intracellular drug accumulation through efflux pump inhibition [9] or by enhanced membrane permeabilization [10], in turn improving the bactericidal activity of the drug, too. For biological applications, it is essential for IONPs to be stabilized with the help of stabilizing agents [11–12]. This helps to reduce the nanoparticle toxicity and facilitates the synthesis of stable nanoparticle dispersions, with reduced size or aggregation [11,13–14]. In this regard, the use of drugs as both a stabilizing agent and an antibiotic could prove to be beneficial. To this end, an understanding of drug–nanoparticle interactions can enable the identification of key parameters for optimal drug loading and drug release, too [15].

Fluoroquinolones, a class of broad-spectrum DNA gyrase inhibiting antibiotics, are used as therapeutics for many intracellular pathogens [16–20]. Recently, they have been explored for their activity as an anti-TB drug [16–17]. Currently, they are used as second-line anti-TB drugs against mycobacteria, which are found to be resistant to rifampicin and/or isoniazid, the first-line drugs [17]. Although at present, moxifloxacin, ofloxacin and levofloxacin are the major fluoroquinolones used in tuberculosis therapeutics [21], many clinical isolates of *M. tuberculosis* have shown resistance towards these drugs, too. Use of iron oxide nanoparticles as drug delivery agents could assist the uptake of drugs and thus, overcome drug resistance [8–10]. Fluoroquinolones are known to form complexes with metal ions through bidentate or unidentate co-ordination bonds [22]. Thus, IONPs, through their surface Fe^2+/3+^ moieties, could exhibit significant drug loading and therefore, have potential as fluoroquinolone delivery agents.

Norfloxacin (NOR), the most basic fluoroquinolone [23] also exhibits anti-mycobacterial activity [24]. It chemically consists of a quinolone carboxylic acid with fluorine and a piperazine ring [25]. It exhibits a zwitterionic nature, with a p*K*a_1_ and p*K*a_2_ of 6.2 and 8.5, respectively [26]. Zwitterionic molecules like amino acids and amphoteric hydroxy groups get adsorbed onto iron oxide nanoparticles predominately via electrostatic interaction [27–28]. Furthermore, their interaction with IONPs may be via carboxylate groups, amine groups or by neither [27]. pH variations thus can play a key role in promoting interactions between amino acids and metal oxide surfaces [29]. NOR has also been reported to form stable complexes with Fe^2+/3+^ [22]. It was also observed in our previous study that IONPs can be loaded with NOR in the absence of stabilizing agents. Drug loading in our previous study was carried out without monitoring pH and was observed to be just 17% [30]. This low drug loading limits the therapeutic applicability of the nanoparticles at high drug concentrations due to toxicity concerns. Thus, we hypothesized that the overall zwitterionic and chelating properties could enable a pH dependent enhanced loading of NOR on to IONPs, independent of any additional stabilizing agent in the formulation, which would in turn permit their application.

In the present work, we used NOR as a model fluoroquinolone and a zwitterionic drug, to explore its interaction with IONPs and further achieve any potential improvement in intra-macrophage delivery and drug accumulation. Being a zwitterionic drug, NOR exists in 3 forms; NOR^+^ (at pH < 6.2), NOR^±^ (at pH 7) and NOR^−^ (at pH > 8.5). We believe that alteration of the coating pH would alter the attraction of the drug to the IONPs, in turn affecting drug loading on IONPs. Thus, we have selected an acidic pH of 5 and an alkaline pH of 10. To further test the drug delivery capacity of the particles, we investigated the drug and nanoparticle uptake in macrophage cells in vitro, as macrophages are the primary site of infection for many intracellular pathogens including *Mycobacterium* [31].

## Results and Discussion

**Figure 1:**
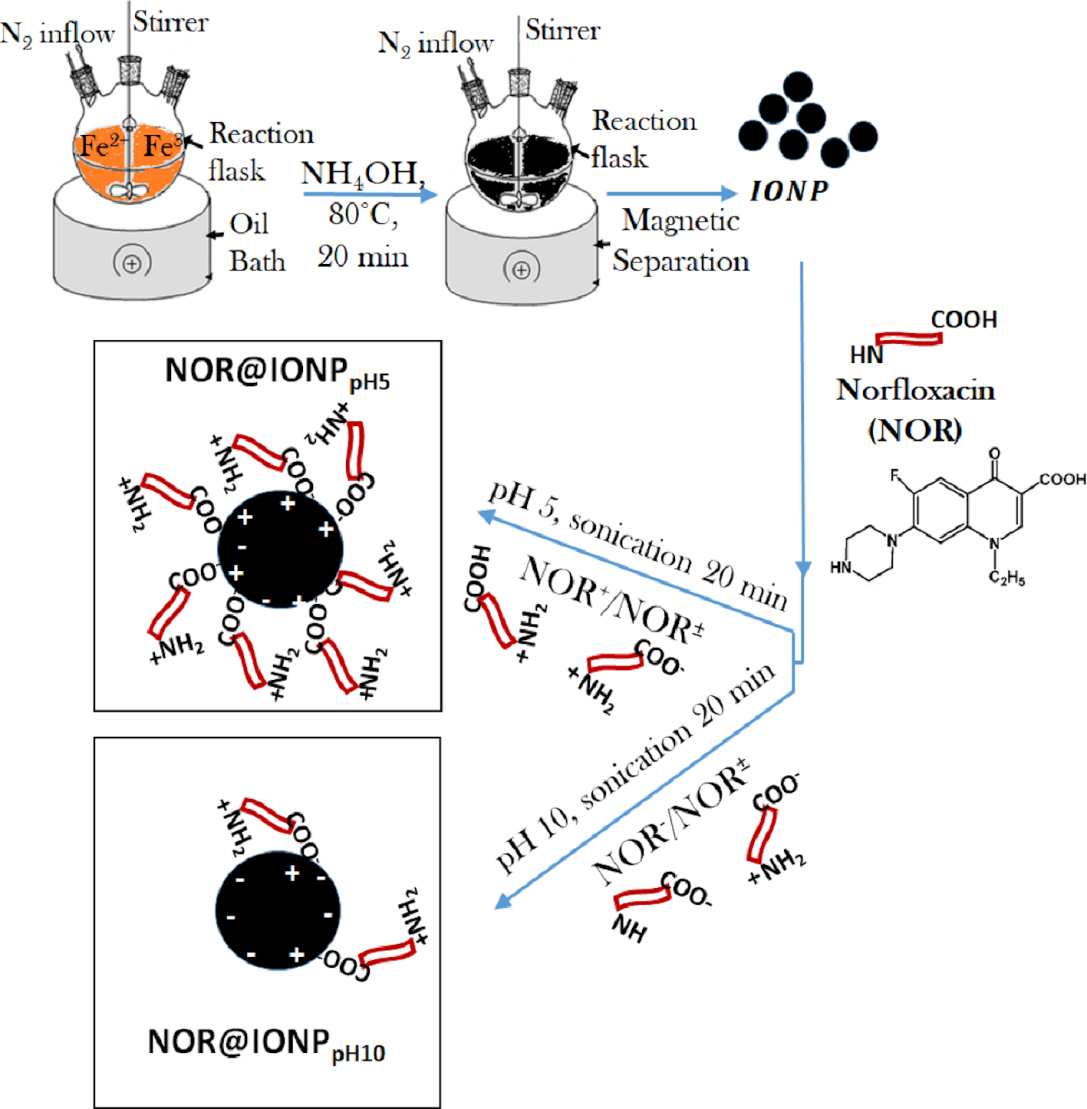
A representation of the iron oxide nanoparticle synthesis followed by the steps for drug loading. NOR@IONP_pH5_ are particles coated with drug at pH 5, showing higher drug loading. NOR@IONP_pH10_ are particles coated with drug at pH 10, showing lower drug loading.

Iron oxide nanoparticles were successfully synthesized, indicated by the appearance of a black coloration upon addition of ammonia solution (Figure 1). NOR was loaded onto the IONPs at pH 5 or pH 10 and the nanoparticles were subsequently analyzed. It was noted that the efficacy of NOR against *Mycobacterium smegmatis* remained unaffected by a change of pH, to either pH 5 or pH 10 (Supporting Information File 1, Table S1).

## Characterization of uncoated iron oxide nanoparticles and NOR

**Figure 2:**
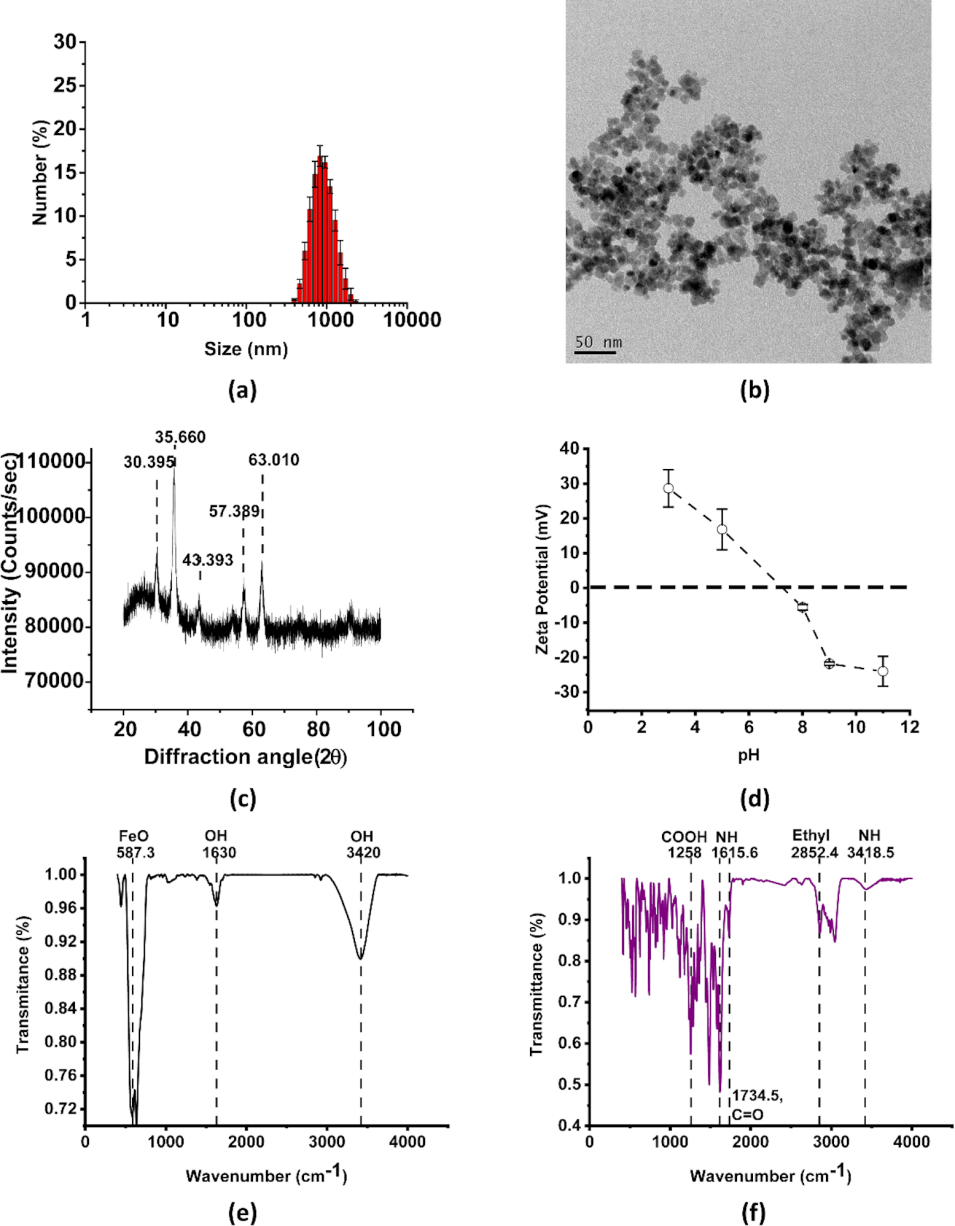
Characterization of uncoated iron oxide nanoparticles using (a) DLS for hydrodynamic diameter (*n* = 3), (b) TEM for individual nanoparticle size estimation (50 nm scale bar), (c) XRD for characterization of the nanoparticle form and crystal structure, (d) zeta potential (error bars indicate standard deviation determined over 3 replicates for each pH) and (e) FTIR spectrum. (f) FTIR spectrum for standard NOR.

Uncoated iron oxide nanoparticles (UIONPs) exhibited a hydrodynamic diameter (from DLS) greater than 1000 nm, which was due to the aggregation of ≈10 nm individual UIONPs, as observed by TEM (Figure 2a,b). The size distribution of these particles was also high, i.e., FWHM of 670.94 nm, resulting from the variation in particle aggregate sizes. The XRD pattern obtained for the synthesized particles were in accordance with the pattern observed in the XRD database for iron oxide (COD: 9013529) [32] (Figure 2c). The zeta potential of UIONPs was found to be dependent on the pH of the dispersion media, varying from positive to negative, as the pH was changed from acidic to alkaline (Figure 2d). The standard deviation for zeta potentials at pH of 8 and 9 was negligible and therefore not discernable in Figure 2d. This is due to the interaction of water molecules with the Fe ions on the surface of IONPs, which in turn facilitates protonation and deprotonation with varying pH [33]. Characteristic iron oxide and NOR peaks were observed in their respective FTIR spectra and were used as a reference for comparison with the coated samples (Figure 2e,f). FTIR peaks observed at 587–590 cm^−1^, 1630 cm^−1^ and 3420 cm^−1^ in Figure 2e correspond to Fe–O vibrations and O–H bending and stretching vibrations, respectively [34]. The O–H vibrations present in the iron oxide nanoparticles possibly arise from the association of oxygen from the aqueous solution to Fe present on the surface of the nanoparticles. Such Fe–OH associations are often found on iron oxide nanoparticles due to their high reactivity [28,35]. Characteristic peaks for NOR observed at 1258 cm^−1^, 1615 cm^−1^, 1734 cm^−1^, 2852 cm^−1^ and 3418 cm^−1^ in Figure 2f are indicative of COOH, NH (quinolone), C=O, CH and NH (piperazine) vibrations, respectively [36].

## Characterization of NOR-coated IONPs, coated at pH 5

**Figure 3:**
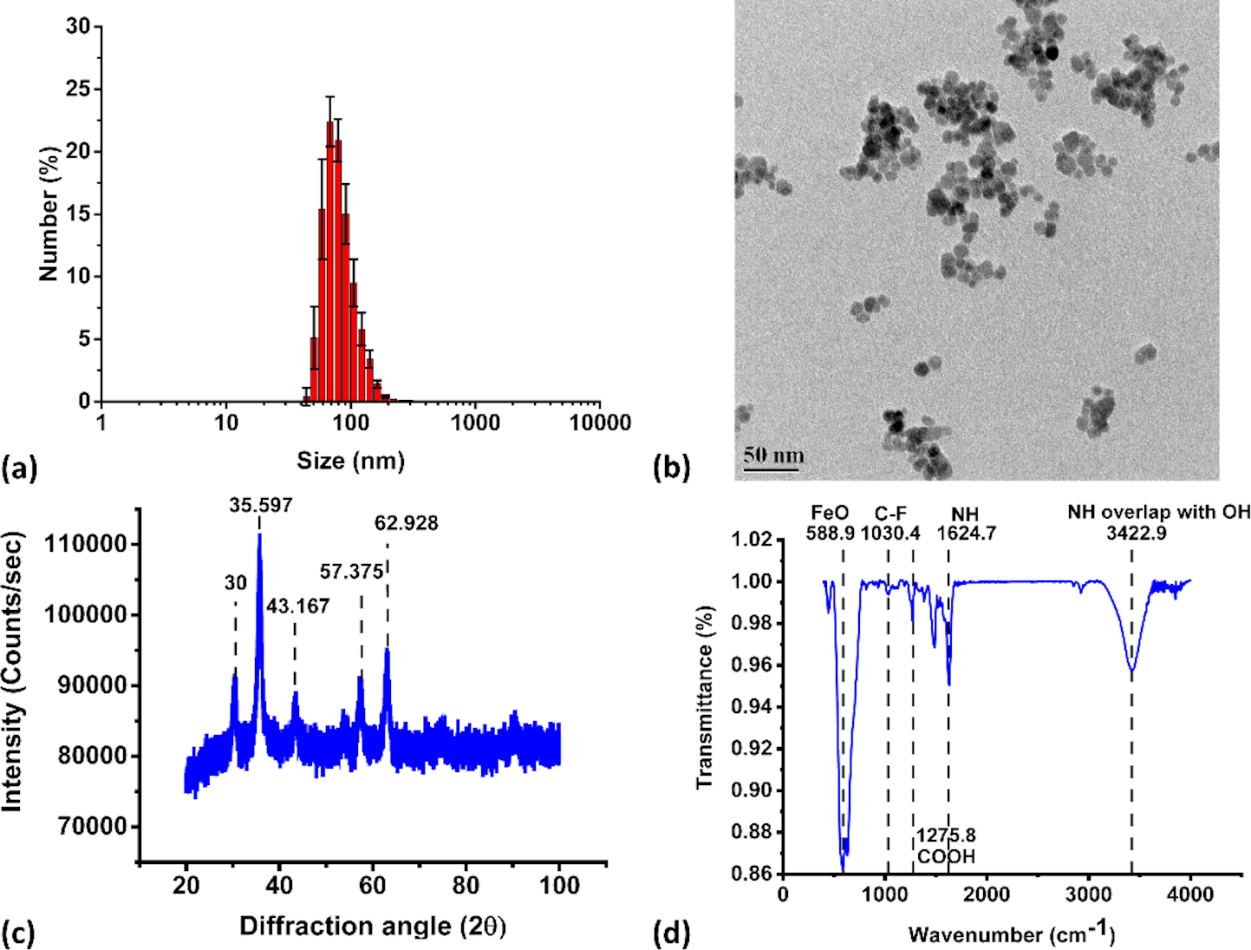
Characterization of NOR@IONP_pH5_ nanoparticles. (a) Dynamic light scattering plot of number (%) v/s size (nm) of the nanoparticles in solution at the end of synthesis (*n* = 3). (b) Transmission electron microscopy image with a 50 nm scale bar. (c) X-ray diffraction plot indicating the Bragg’s 2θ diffraction angles identical to peaks observed for crystalline iron oxide. (d) FTIR spectrum of transmittance v/s wavenumber, depicting major functional groups of iron oxide and NOR.

NOR@IONP, coated at pH 5 (NOR@IONP_pH5_), exhibited a distribution size range of 45 to 110 nm (Figure 3a), which was confirmed by TEM, to be aggregates of 10–12 nm size individual nanoparticles (Figure 3b), clearly indicating a reduced aggregate size in comparison to the UIONPs which was also indicated by the reduced FWHM, i.e., 40.72 nm. The XRD pattern confirmed the presence of iron oxide nanoparticles (Figure 3c). The FTIR spectrum of NOR@IONP_pH5_ indicated the presence of Fe–O stretching, from the IONPs, along with characteristic peaks of amines. C–F, N–H bending of quinolones and N–H stretching of piperazinyl were observed at 550–650, 1000–1050, 1650 and 3300–3500 cm^−1^, indicating the presence of NOR (Figure 3d) [36–38]. It was noted that the N–H stretching of piperazinyl and O–H lie within the same wavenumber range, thus, the peak observed at 3422.9 cm^−1^ could be attributed to both NH from NOR and OH from the surface Fe–OH groups present in the sample. Fluoroquinolones are believed to chelate Fe ions through their carboxylate and amine groups [22]. The FTIR peak shifts observed at 1258 cm^−1^ (COOH stretching) and 1615.6 cm^−1^ (NH bending) to higher wavenumbers could possibly be due to such interactions of NOR with Fe^2+/3+^ existing on the surface of iron oxide nanoparticles [39–40]. This was also confirmed by the disappearance of the C=O stretching vibration at 1734.5 (Supporting Information File 1, Figure S1). Such peak shifts are also observed when NOR interacts with metal ions like NiO [41]. The zeta potential of these particles was found to be +29 mV, validating the loading of drug and indicating nanoparticle stability at neutral pH.

## Characterization of NOR-coated iron oxide nanoparticle coated at pH 10

**Figure 4:**
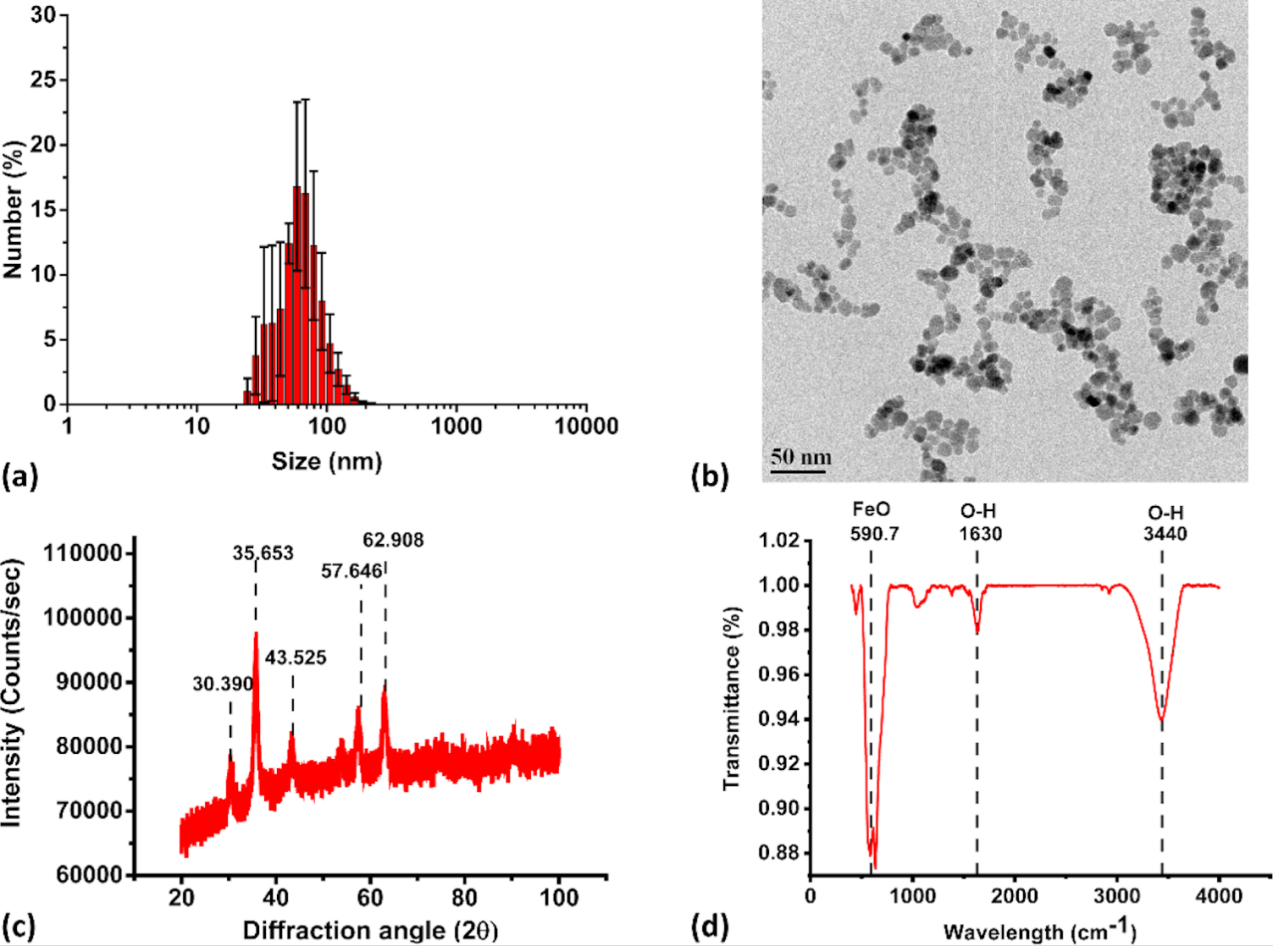
Characterization of NOR@IONP_pH10_ nanoparticles. (a) Dynamic light scattering plot of number (%) v/s size (nm) of the nanoparticles in solution at the completion of synthesis (*n* = 3). (b) Transmission electron microscopy image with a 50 nm scale bar. (c) X-ray diffraction pattern indicating the Bragg’s angle corresponding to those observed in iron oxide. (d) FTIR plot of transmittance v/s wavenumber depicting functional groups of iron oxide.

NOR@IONP coated at pH 10 (NOR@IONP_pH10_), had a size distribution ranging from 25 to 120 nm, as examined through DLS (Figure 4a). TEM further confirmed these to be aggregates of 10–13 nm sized individual nanoparticles (Figure 4b). The FWHM was also observed to be 46.97 nm exhibiting a narrower size distribution in comparison to UIONPs but a slightly broader in comparison to NOR@IONP_pH10_. Thus, coating at pH 10 also promoted a reduction in the nanoparticle aggregate size. A slight shoulder in the hydrodynamic size distribution at 32–40 nm is not a true peak and the distribution is unimodal. The hydrodynamic and TEM data however, do indicate the presence of a fraction of smaller sized particles in comparison to even NOR@IONP_pH5_. This could be due to a greater charge on the nanoparticles during drug coating which possibly reduces the particle aggregation. The XRD pattern of NOR@IONP_pH10_ was indicative of the presence of iron oxide nanoparticles (Figure 4c). Further confirmation of iron oxide was found through the Fe–O stretching, observed at 590 cm^−1^ in the FTIR spectrum [37–38]. The FTIR spectrum of the sample indicates either the absence of NOR or its presence in very minute quantities (Figure 4d). A shift observed in the FTIR peak for OH stretching from 3420 cm^−1^ to 3440 cm^−1^ (Supporting Information File 1, Figure S2) could be a result of changes in the intermolecular H bonding, whereby we believe that a fraction of the hydrogen bonding in NOR@IONP_pH10_ could occur through the amine groups present in NOR and the negatively charged IONPs at pH10 during drug coating. This could be due to the occurrence of negative species of both IONP and NOR at pH 10, resulting in a large electrostatic repulsion and consequent low drug loading. The zeta potential of these particles was indeed found to be −16.5 mV, supporting this.

Comparing the different nanoparticles synthesized, i.e., UIONPs, NOR@IONP_pH5_ and NOR@IONP_pH10_, we observed that the drug coated IONPs have a much lower aggregate size with a reduced hydrodynamic size distribution (Table 1). The NOR@IONPs also carry a surface charge and neutral pH. Thus indicating that these particles indeed have an improved stability, possibly due to the coating of drug. NOR@IONPpH5 also appear to have a higher drug loading.

**Table 1:** Hydrodynamic diameter, the FWHM and size distribution of respective UIONP, NOR@IONP_pH5_ and NOR@IONP_pH10_ samples.

Sample	Mean nanoparticle size as per DLS (nm)	FWHM (nm)	Size distribution (FWHM/mean)

UIONP	952.9	670.94	70.4%
NOR@IONP_pH5_	77.3	40.72	52.7%
NOR@IONP_pH10_	66.10	46.97	71.1%

It was noted that both NOR@IONP_pH5_ and NOR@IONP_pH10_ had 2–3 nm larger individual particle size in comparison to the NOR@IONPs synthesized in our previous study. However, the hydrodynamic diameter in this work is observed to be smaller, with majority of the particles lying between 70–80 nm and 40–70 nm for NOR@IONP_pH5_ and NOR@IONP_pH10_, respectively. Additionally, the zeta potential resembled that of NOR@IONP_pH5_ but the drug loading achieved was 3 times lower than that achieved in the NOR@IONP_pH5_ system of this study (Table 2). Thus, NOR@IONP_pH5_ resembled the NOR@IONPs from our previous study in the surface potential but differed in size.

**Table 2:** Comparison of different NOR@IONPs synthesized in previous and current studies.

Nanoparticle sample	Individual size (nm) (TEM)	Hydrodynamic size (nm) (DLS)	Zeta potential (mV)	Drug coating (µg/mg of nanoparticle)	Reference

NOR@IONP	8.87	200	28.5	17.13	[30]
NOR@IONP_pH5_	12.9	78.8	29	50.2	This study
NOR@IONP_pH10_	11.3	66.7	−16.5	6.5	This study

## Drug release and coating estimation

**Figure 5:**
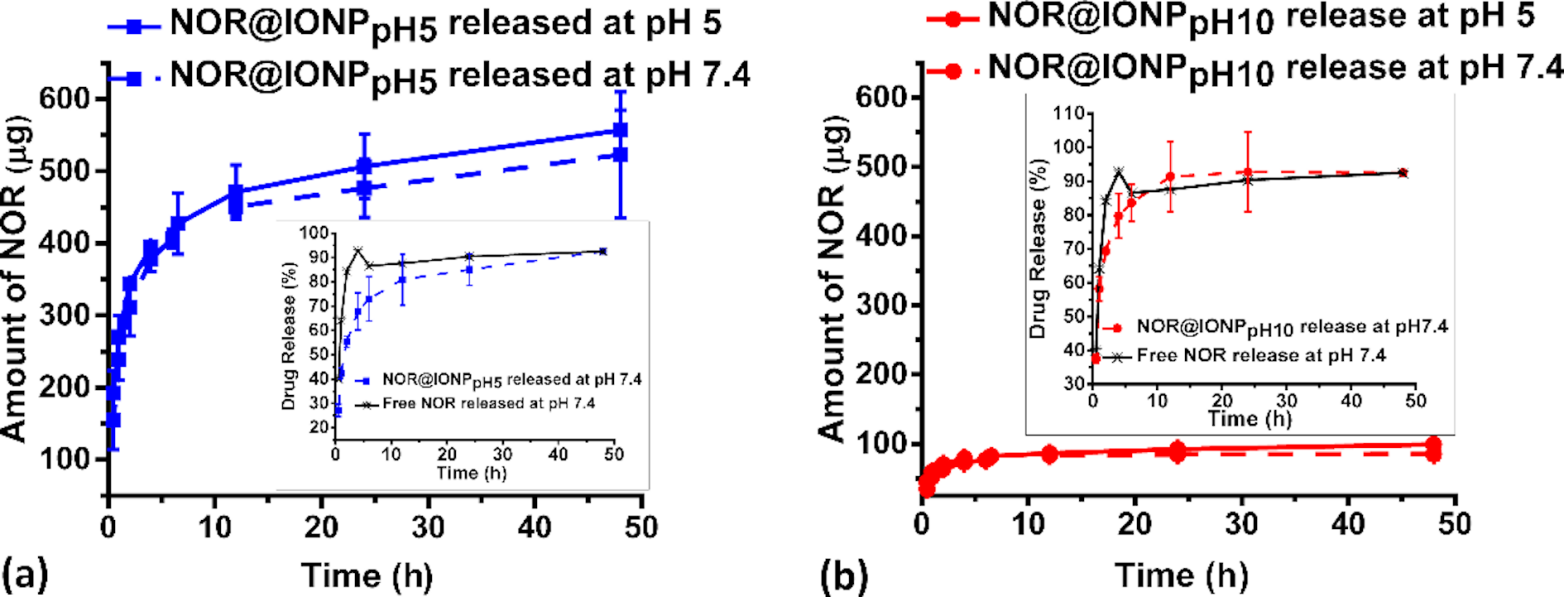
Drug release profile over 48 h (a) from NOR@IONP_pH5_ and (b) from NOR@IONP_pH10_, where solid lines indicate a release in pH 5 medium, while the dashed lines indicate a release in a medium with pH 7.4. Insets depict percentage drug release for comparing the release of free drug and drug coated on nanoparticles (solid lines – for free drug, dashed lines – for nanoparticles). The standard deviation plotted for each data point is obtained from 3 replicates each.

Intracellular pathogens are often contained in vesicles within phagocytic cells like the macrophages. These vesicles are known to present an acidic environment [42], while the normal physiological pH of the cell remains neutral [43]. Thus, nanoparticles used in drug delivery to macrophages would experience both neutral (pH 7.4) and acidic (pH 5) conditions. To investigate any alterations in the drug release profile from the NOR@IONPs based on the cellular pH variations, the drug release was monitored over 48 h in phosphate buffer saline (PBS) release media, maintaining the pH at either 5 or 7.4. We observed that the release kinetics and saturation amounts were identical in both the pH (5 or 7.4) of the release media, irrespective of whether NOR@IONP_pH5_ or NOR@IONP_pH10_ was used (Figure 5). Free drug is observed to have rapid release profile which saturates by 4 h. NOR@IONP_pH5_ displays an initial burst release up to 1 to 2 h after which a slow release of NOR is observed. This initial burst release could arise from weakly interacted NOR in the surface of the nanoparticles, while a followed slow release could arise from more strongly interacting NOR on the nanoparticles (Figure 5a, inset). The drug release profile from NOR@IONP_pH10_ resembled that of free NOR where the release was rapid over the first 4–6 h and saturated out there after (Figure 5b, inset). The release of NOR from metal oxides, NiO, is found to follow first order rate kinetics thus we too fitted out drug release plots to a first order model [41]. The drug release rate constants were calculated to be 1.3, 0.7 and 1.1% h^−1^ for free NOR, NOR@IONP_pH5_ and NOR@IONP_pH10_, respectively (Supporting Information File 1, Figure S3a). Thus confirming that a slower drug release rate for NOR@IONP_pH5_. This release profile was also observed to be in accordance with the previously synthesized NOR@IONPs, where it was observed that NOR is released rapidly over the first 3–4 h but is slow and sustained after 4 h [30]. Additionally, the rate constant obtained for previously synthesized (non-pH characterized) NOR@IONPs was estimated to be 0.47% h^−1^ which is slightly reduced in comparison to the NOR@IONP_pH5_ system as well (Supporting Information File 1, Figure S3c). Thus, NOR@IONP_pH5_ enables a slow drug release although the rate of release is marginally greater than the NOR@IONPs in our previous work.

The estimated drug coating for NOR@IONP_pH5_ and NOR@IONP_pH10_ were in the range 50.2 ± 7.4 (mean ± standard deviation) µg/mg of nanoparticle and 6.5 ± 2.1 µg/mg of nanoparticle, respectively. Thus, confirming that a greater drug–nanoparticle attraction occurs at acidic coating pH of 5, which in turn enhances drug loading on IONPs, even in the absence of any extraneous linker molecules. The low drug loading at a coating pH of 10, could be verified by the absence of any prominent NOR spectral peaks in the FTIR spectrum of NOR@IONP_pH10_, as described earlier (Figure 4d). It was also noted that the drug coating achieved through acidification of the medium during drug loading is 3 times greater than determined for NOR@IONPs synthesized in our previous study, where the drug coating was estimated to be 17.13 µg/mg of the nanoparticle [30].

At pH 5, we know from the zeta potential that, IONPs express a positive charge (Figure 2d). Therefore, an electrostatic interaction would occur between IONP and the negatively charged carboxylate group of NOR, as carboxylate would be present on the zwitterionic NOR^±^ molecule at this acidic pH. The percentage of zwitterionic NOR^±^ form, estimated through the Henderson–Hasselbalch equation is only 5.9% (Supporting Information File 1, Table S2) [29,44–45], with the positively charged NOR^+^ form being the dominant rest amount of about 94%. So, IONP cannot electrostatically interact with NOR^+^ at this pH 5 and hence more NOR would not have coated on IONP, too. However, in spite of this, the coating efficiency achieved at pH 5 ranged as high as 43–51%, which is much greater than the percentage of NOR^±^ at this pH (Supporting Information File 1, Table S3). This is possible because NOR contains other electronegative groups, like fluoride, or π–π electron rich regions (quinolone ring and ketone), which can interact with positively charge IONP at pH 5. This in turn has resulted in the observed enhanced drug coating efficiency of 43–51% (Supporting Information File 1, Figure S5) [28].

On the contrary, at pH 10, as per zeta potential, IONP has a negative charge and can only interact with the positive part of the zwitterionic NOR^±^. However, the percentage of NOR^±^ is only 3.1% at pH 10 (Supporting Information File 1, Table S2). In fact, the coating efficiency also ranges from 4.9–9.2% (Supporting Information File 1, Table S3) [29,44]. This is statistically identical to the 3.1% zwitterionic fraction of NOR (NOR^±^), at pH 10. This again indicates that, at pH 10, the drug-nanoparticle interaction is only through the electropositive (–NH) group present in NOR^±^ (Supporting Information File 1, Figure S5). Furthermore, as NOR does not contain any additional electropositive groups in its structure, hence no further enhancement in drug coating can be achieved at pH 10 (unlike as in pH 5); it remains low, as measured in the experiments.

## Drug delivery application of NOR@IONPs

**Figure 6:**
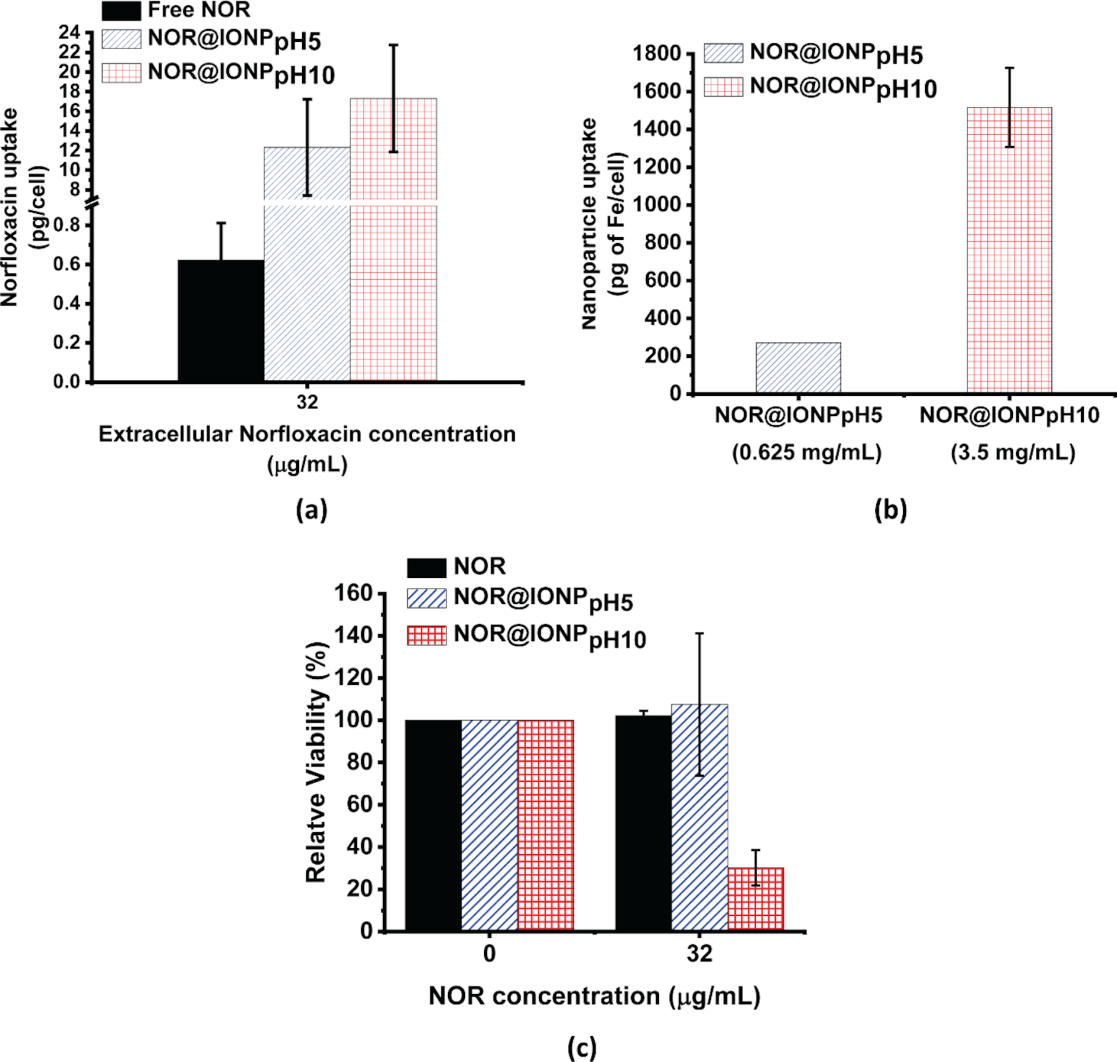
(a) NOR uptake in macrophage cells after 48 h treatment with NOR, extracellular concentration of 32 µg/mL. (b) Nanoparticle uptake estimated in terms of the uptake of Fe (pg/cell). (c) Relative viability of differentiated THP1 cells after treatment with 32 µg/mL of NOR over 48 h. The black columns depict free drug treatment, blue diagonal patterned columns depict treatment via NOR@IONP_pH5_ and red checked columns depict treatment with NOR@IONP_pH10_. Statistical significance was estimated using the student’s *t*-test where “***” represents α ≤ 0.001. All data is plotted as the mean and standard deviation of 3 biological replicates.

The efficacious concentration of NOR (in RPMI media supplemented with 10% fetal bovine serum (FBS)) against extracellular *M. smegmatis* is 8 µg/mL (Supporting Information File 1, Figure S6). To enable an intra-macrophage bacterial clearance however, a higher NOR concentration would be required. Therefore, to investigate the use of nanoparticles for the drug delivery in macrophage cells, a NOR concentration that exceeds 8 µg/mL (i.e., 32 µg/mL) was selected. When treated with an extracellular NOR concentration of 32 µg/mL, the NOR uptake in macrophage cells was found to be 0.3 pg/cell over 48 h. A drug delivery via NOR@IONP_pH5_ and NOR@IONP_pH10_ at extracellular NOR concentration of 32 µg/mL resulted in increased uptake of 7 and 12 pg/cell, respectively, over identical treatment conditions (Figure 6a). The increased uptake could be due to active engulfment of nanoparticles by macrophages, due to their larger size (20–120 nm size range) and surface charge (+29 mV or −16.5 mV) [46], which results in simultaneous internalization of larger amounts of drug. Figure 6b shows that the nanoparticles are also taken up efficiently by the macrophages. Additionally, a higher uptake of NOR@IONP_pH10_ nanoparticles is entirely because of the higher amount of nanoparticles required to achieve identical drug concentrations, a result of low drug loading. To achieve an extracellular NOR concentration of 32 µg/mL, the nanoparticle concentration of NOR@IONP_pH5_ and NOR@IONP_pH10_ was 0.625 and 3.5 mg/mL, respectively (Figure 6b). Thus, via the NOR@IONP_pH5_ nanoparticle system, a 22-fold increase in the intra-macrophage NOR concentration was achieved, even at a lower nanoparticle concentration. The drug uptake is also greater than the uptake observed through our previous work, where the NOR@IONPs enhance the drug uptake of only 7-fold. Furthermore, the relative viability of NOR, NOR@IONP_pH5_ and NOR@IONP_pH10_ for 32 µg/mL drug concentration are found to be 102.1, 107.5 and 30.1%, respectively. Thus, both NOR and NOR@IONP_pH5_ administered at this concentration, exert no toxicity towards the macrophage cells. This is in accordance with our previous study [30] on macrophages, where we reported that the NOR becomes toxic at concentrations greater than 100 µg/mL, while the toxicity of IONPs greatly increases above concentrations of 1 mg/mL. It was also noted that the NOR@IONP_pH5_ exhibit reduced toxicity in comparison to NOR@IONPs from our previous study which exhibited a relative viability of 50% when administered at a NOR concentration of 32 µg/mL (data not shown) [30].

**Figure 7:**
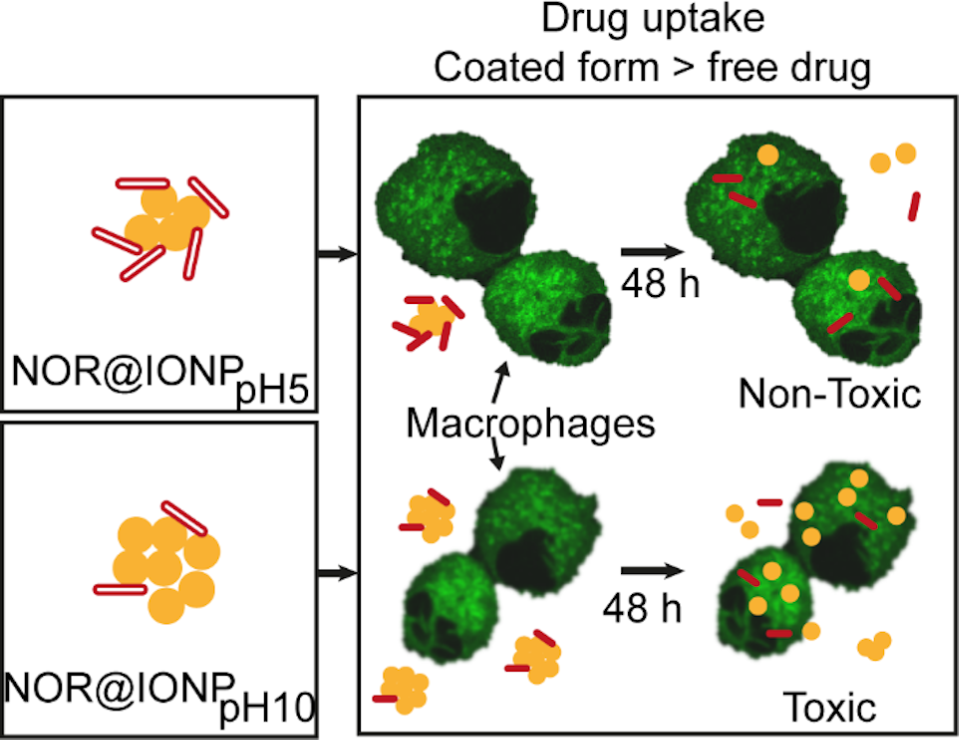
Schematic showing the effect of NOR@IONPs, coated at either pH 5 or at pH 10, on its role in drug and nanoparticle uptake in macrophage cells. Yellow circles represent the IONPs while red rods represent the drug (NOR). The macrophage cells are in green with black regions depicting unstained regions in the cell.

We therefore concluded that the NOR@IONPs enhance drug uptake in comparison to the free drug. In the case of NOR@IONP coated at pH 10, however, the large amount of nanoparticle required for achieving the desired extracellular drug concentration may not be useful for application (Figure 7). In our present study, NOR@IONP_pH5_ however, attains the desired extracellular drug levels with a much lower nanoparticle concentration (0.625 mg/mL), thus proving it to be non-toxic.

Since increasing the drug loading (achieved in case of NOR@IONP_pH5_) enables the use of a reduced nanoparticle concentration (to reach the desired extracellular drug concentration), the limitation imposed by the nanoparticle toxicity is overcome by the NOR@IONP_pH5_ system. Thus, the use of pH 5 for drug loading onto IONPs provides the desirable additional benefit of the reduced toxicity towards macrophage cells.

## Conclusion

The general inability to increase the intracellular drug concentration in macrophages (having engulfed pathogens, like *Mycobacterium*) results in high drug dosage requirement for the pathogen clearance. This is a major hurdle in tuberculosis treatment. In turn, a high dosage causes toxic side effects from either the drug or the carrier nanoparticle, necessitating new delivery systems to enhance the intracellular antibiotic concentration and to avoid toxicity. Furthermore, the choice of linkers and stabilizing agents can also elicit toxicity. In an attempt to overcome these limitations of low drug loading and toxicity of stabilizing agents, we studied the ability of a zwitterionic drug to coat iron oxide nanoparticles, while also enhancing the particle stability. In this regard, two different NOR-coated IONP systems (NOR@IONPs) were synthesized – namely with a drug coating at pH 5 or at pH 10, respectively. These nanoparticles were stable in aqueous dispersion, due to electrostatic repulsion from the existing charge on their surfaces. We find that, compared to pH 10, an acidic pH of 5 enhances the drug coating on IONPs, in the range of 4.7 to 5.7 times, achieving a NOR loading efficiency almost equivalent to polymeric nanoparticles composed of poly (3-hydroxybutyrate) [41]. This high drug loading was also reflected by the presence of prominent NOR peaks in the FTIR spectrum of NOR@IONP_pH5_, compared to that in NOR@IONP_pH10_. Moreover, as desired in a formulation, the rate of drug release from NOR@IONP_pH5_ over the initial 4 hours was slower than the release of the free drug, while the drug release from NOR@IONP_pH10_ was identical to that of the free drug. So, a combination of higher drug loading and slower release profile indicates a beneficial increased attraction between NOR and IONPs at the lower coating pH of 5. This enhanced interaction, we believe, is due to the electronegative nature of the drug, which facilitates the interaction with positively charged IONPs at an acidic pH. Furthermore, considering the similarity in structure and basic chemical composition of fluoroquinolones, we expect this to be applicable to the other antibiotics of this class, like ciprofloxacin and moxifloxacin.

The quantification of the intra-macrophage accumulation of NOR shows that, cellular uptake is greatly facilitated by drug coated systems too, compared to free drug. Although both drug-coated nanoparticle systems enhance the drug uptake, due to lower drug loading in case of NOR@IONP_pH10_, a higher concentration of nanoparticles would be required to achieve the same extracellular drug concentration. This is disadvantageous, as the higher concentration of iron oxide will induce toxicity in macrophage cells [30].

Thus, our study shows that, zwitterionic drugs can serve as both stabilizing agents and antibiotics, for drug delivery via iron oxide nanoparticles. Additionally, a mere adjustment of the drug coating pH to 5, can greatly enhance drug loading and achieve slower drug release, too. Interestingly, however, irrespective of the drug coating pH, the amount of drug released is independent of the pH of the release medium. NOR@IONP_pH5_ nanoparticles thus constitute a stable iron oxide nanoparticle system, with higher drug loading, slower drug release, reduced toxicity and enhanced uptake in macrophage, which can lead to its application in the treatment of intracellular pathogens.

## Experimental

### Reagents

FeCl_2_·4H_2_O, FeCl_3_·6H_2_O and NOR used in the nanoparticle synthesis were purchased from Sigma-Aldrich, Germany. 25% ammonia solution was purchased from Merck, Germany (GR grade).

RPMI-1640 and fetal bovine serum (FBS) used for the culturing of THP1 cells were purchased from HiMedia, India. Phorbol 12 myristate acetate (PMA) used for THP1 cell differentiation was purchased from Sigma-Aldrich, Germany.

### Synthesis

The synthesis of IONPs and NOR@IONPs was carried out analogously to our previous study [30]. Specifically, IONPs were synthesized by co-precipitation of ferrous and ferric chloride salts [47]. An aqueous solution of Fe^2+^:Fe^3+^, taken in the molar ratio of 1:2, was stirred at 700 rpm with nitrogen purging, for 10 min, at 80 °C. The reduction to Fe_3_O_4_ was carried out with the addition of 15 mL of 25% ammonia solution. Stirring and reaction was continued for 20 min more, at 80 °C. The dispersion was then allowed to cool, and the nanoparticles were magnetically separated out and washed with milliQ water. IONPs synthesized from 100 mL reaction was dispersed in 100 mL milliQ water and coating of NOR was carried out with a solution of 1 mg/mL drug concentration. The dispersion of nanoparticles and drug was adjusted for pH, to allow coating at either pH 5 or pH 10, while being sonicated for 20 min. This step was added in order to ensure pH monitoring for drug coating and was not present in our previous work. A step involving the stirring of the coated nanoparticle suspension at 80 °C was omitted in this work. Finally, the coated nanoparticles were separated from non-adsorbed drug, by centrifugation at 10,000 rpm for 1–2 h (centrifugation speed and time were increased in comparison to our previous work in order to facilitate the settling of smaller sized nanoparticles synthesized in this work). The pellet was oven-dried at 60 °C and then powdered using a mortar and pestle. The desired amount of the particle was re-suspended in water, by probe-sonication for 30 s (50% amplitude, 2 s on, 2 s off pulse) (Sonics, Vibra Cell, USA). NOR@IONP synthesis, at each of the respective pH, were performed with 3 distinct replicates.

### Nanoparticle characterization

The hydrodynamic diameter of nanoparticles was determined after re-dispersing in milli-Q water and loading the sample in a cuvette for dynamic light scattering (DLS) measurements using a ZetaSizer (Malvern Instruments, UK). The refractive index and temperature used for the size measurement was 2.34 and 25 °C, respectively. Three replicates were measured for each sample. In addition, the nanoparticle diameter was also measured from transmission electron microscopy (TEM) images, which were obtained using the JOEL-JEM 2100F TEM 200 kV, USA. For TEM imaging, re-dispersed nanoparticle samples were diluted and 10 µL was loaded on a formvar coated Cu grid. The sample was dried using an infrared lamp. The images obtained were analyzed using the ImageJ software.

X-ray diffraction (XRD) was obtained by loading powdered nanoparticle samples for analysis in the PANalytical, X’Pert Pro, UK, facilitated with a Cu Kα radiation source. The Fourier transform infrared (FTIR) spectra of the nanoparticles and drug were collected using a 3000 Hyperion Microscope with a Vertex 80 FTIR system. Nanoparticles re-dispersed in milli-Q water were loaded into a folded capillary cuvette and the zeta potential was measured using the NanoS Zeta Sizer, Malvern Instruments, UK.

### Drug coating and release

NOR coating on the nanoparticle was estimated via drug release, where it was assumed that at 48 h, complete drug release occurs, while the equilibrium is achieved between the drug concentration within the dialysis bag and that in the release media. This equals 92.5% of drug release. The drug release was monitored over 48 h, by sampling 1 mL of release medium at each time point and subsequently correcting the volume reduction due to sampling loss. The concentration of NOR released was estimated fluorometrically (Ex/Em: 280/420 nm) (SpectraMax M5, Molecular Devices) using a known NOR concentration versus fluorescence standard, prepared with dilution in phosphate buffer saline (PBS) (same as the release medium).

### Drug and nanoparticle uptake in macrophages

THP1 cells, a monocyte cell line, was differentiated into macrophage cells through a treatment with 25 ng/mL of phorbol 12-myristate acetate (PMA), for 24 h, at 37 °C and 5% CO_2_. Post incubation, PMA was washed off and fresh RPMI-1640 supplemented with 10% FBS was added and the cells were allowed to stabilize for 24 h under identical conditions of temperature and CO_2_. Differentiation was confirmed by the visualization of adhered cells to the bottom of the culture well, as the THP1 monocytes are suspension cells. The differentiated THP1 cells, i.e., the macrophages, were then treated with 32 µg/mL of NOR, in either free or nanoparticle-coated form, for 48 h. The extracellular drug and nanoparticles were washed off with ice cold PBS and cells lysed overnight, with 0.1 N glycine–HCl, at pH 3.5. A fixed volume of each cell lysate was sampled for ICP-AES (inductively coupled plasma-atomic emission spectrophotometer) (ACROS, Simultaneous ICP Spectrometer, SPECTRO Analytical Instruments GmbH, Germany) to estimate the amount of Fe present in each sample. Cell lysates were pelleted out and the supernatant was used for spectrofluorometric estimation of intracellular NOR (Ex/Em: 280/420 nm) (SpectraMax M5, Molecular Devices). The NOR standard curve in this study was prepared by diluting the drug in 0.1 N glycine-HCl, pH 3.5.

### Toxicity

Monocyte, THP1 cells were seeded at 3 × 10^5^ cells/mL and differentiated into macrophages by treating with 25 ng/mL PMA for 24 h. Differentiated THP1 cells (macrophages) were stabilized in fresh RPMI media, supplemented with 10% FBS for 24 h at 37 °C and 5% CO_2_. These macrophage cells were subsequently treated with 32 µg/mL of NOR, either in its free drug form or via NOR@IONP, for 48 h. Following the drug/nanoparticle treatment the cells were trypsinized using 1X trypsin-EDTA (HiMedia, India). The cell counts of the trypsinized cells were then determined using the trypan blue assay. Briefly, a fixed volume of animal cell sample was taken and diluted using a 0.4% solution of trypan blue (HiMedia, India). The diluted cell suspension was then gently vortexed and 10 µL loaded on to a hemocytometer for cell counting. The relative viability of the sample was determined with respect to the non-treated control.

## Supporting Information

File 1 Supplementary Information.
Data that supports the experimental choices and data analysis.
