# Supplementary material for: pH-driven enhancement of anti-tubercular drug loading on iron oxide nanoparticles for drug delivery in macrophages
Source: Beilstein J Nanotechnol. 2021 Oct 7;12:1127–39. doi: 10.3762/bjnano.12.84 (PMC8505898; doi:10.3762/bjnano.12.84)
Supplement: File 1 — Supplementary Information. Data that supports the experimental choices and data analysis. [file Beilstein_J_Nanotechnol-12-1127-s001.pdf]

BEILSTEIN JOURNAL OF NANOTECHNOLOGY

# Supporting Information

for

# pH-driven enhancement of anti-tubercular drug loading on iron oxide nanoparticles for drug delivery in macrophages

Karishma Berta Cotta, Sarika Mehra and Rajdip Bandyopadhyaya

*Beilstein J. Nanotechnol.* **2021,** *12,* 1127–1139. doi:10.3762/bjnano.12.84

## Supplementary Information.

## Data that supports the experimental choices and data analysis

License and Terms: This is a supporting information file under the terms of the Creative Commons Attribution License (https://creativecommons.org/licenses/by/4.0). Please note that the reuse, redistribution and reproduction in particular requires that the author(s) and source are credited and that individual graphics may be subject to special legal provisions.
The license is subject to the *Beilstein Journal of Nanotechnology* terms and conditions: (https://www.beilstein-journals.org/bjnano/terms)

**Zone of inhibition assay to investigate the effect of pH on the efficacy of NOR**

*M. smegmatis* cells cultured to an optical density at 600 nm ($OD_{600}$) of 0.5, were spread plated on Luria Bertani Agar. Wells were bored into the agar at distinct locations. 4 µg of NOR was added into the wells, either from a standard NOR stock solution or from NOR pH 5 stock solution or from NOR pH 10 stock solution. The plates were incubated at 37 °C for 48 h, after which clearance zones around the well were measured in mm. The diameter of the clearance zone is directly proportional to the efficacy of the drug. Thus, an identical zone size obtained for all drug treatments in indicative of unaltered efficacy [Table S1]. This implies that both pH 5 and 10 do not adversely affect the anti-mycobacterial properties of NOR.

**Table S1:** Comparison of the diameter of the clearance zones obtained for *M. smegmatis* treated with 4 µg of NOR as standard, pH 5 or pH 10 solutions.

| **Drug sample** | **Diameter of the zone of inhibition (mm) (mean ± standard deviation)** |
|---|---|
| NOR standard | 14.3 ± 0.6 |
| NOR (pH5) | 14.0 ± 1.0 |
| NOR (pH10) | 13.7 ± 0.6 |

**Calculation for amounts of ionic and zwitterionic forms of norfloxacin (NOR)**

Calculation of absolute percentages of the zwitterionic form of the NOR ($NOR^{\pm}$), at both pH 5 and pH 10 were done through Eq. S3 (given here), with inferences from the Henderson-Hasselbalch equation [Table S2]. Eq. S1 is applicable at $pH \leq pKa_1$ (e.g. at pH 5), while Eq. S2 is applicable for $pH \geq pKa_2$ (e.g. at pH 10).

$$pH = pKa_1 + log\frac{[NOR^{\pm}]}{[NOR^{+}]} \quad (S\ 1)$$

$$pH = pKa_2 + log\frac{[NOR^{-}]}{[NOR^{\pm}]} \quad (S2)$$

$$NOR^{\pm}\ \% = \frac{[NOR^{\pm}]}{[NOR^{\pm}] +\ [NOR^{+\ or-}]} \quad (S3)$$

**Table S2:** Calculated zwitterionic NOR$^{\pm}$ percentages at pH 5 and pH 10.

| **pH** | **pKa** | **NOR$^{\pm}$ %** | **Equation** |
|---|---|---|---|
| 5 | 6.2 | 5.9 | Eqs. S1 and S3 |
| 10 | 8.5 | 3.1 | Eqs. S2 and S3 |

The above Table shows the relatively small percentage amounts of NOR$^{\pm}$ existing in a solution of the drug, namely 5.9 and 3.1%, at pH 5 and 10, respectively. This implies that the majority of the rest of the form of the NOR drug would be the cationic ($[NOR^{+}]$) and the anionic ($[NOR^{-}]$), at pH5 and 10, respectively.

In using the above equations and calculations, we have assumed that the amount of drug added [in a nanoparticle dispersion (IONP, in this case)], is not in excess of the maximum amount of drug that can get adsorbed/coated on the nanoparticles in dispersion, and therefore does not surpass the amount required for considering coating efficiency. This enables us to correlate the coating efficiency [Eq. S4] obtained with percentage of NOR$^{\pm}$ at the respective pH of 5 or 10.

$$Coating\ efficieny\ (\%) = \frac{Total\ drug\ coated}{Total\ drug\ used\ for\ coating} \times 100 \qquad (S4)$$

**Table S3:** Coating efficiencies obtained for NOR@IONPs at pH 5 and pH 10.

| Sample name and its replicate no. | | Total drug actually coated on IONP (mg) | Total drug taken in dispersion for coating purpose (mg) | Coating efficiency (%) |
|---|---|---|---|---|
| $NOR@IONP_{pH5}$ | 1 | 43 | 100 | 43 |
| | 2 | 51 | 100 | 51 |
| $NOR@IONP_{pH10}$ | 1 | 4.9 | 100 | 4.9 |
| | 2 | 9.2 | 100 | 9.2 |

Comparison of low percentages of zwitterionic form at both pH 5 and 10 (in Table S2), with the high and low coating efficiencies at pH 5 and 10, respectively (in Table S3), helps us conclude on the nature of interaction between IONP and drug. This also explains the desirable high drug coating efficiency at pH 5, which is discussed in the main body of the paper.

**FTIR Spectral data zoomed in**

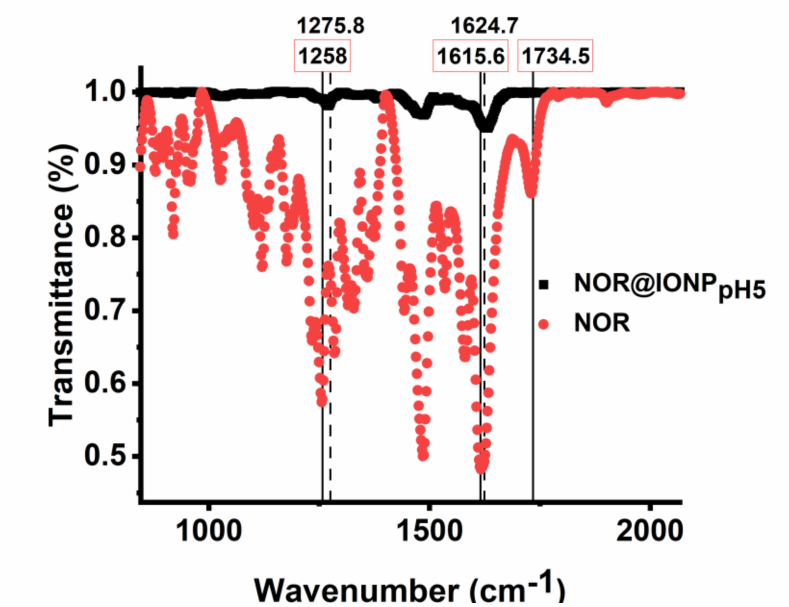


**Figure S1:** FTIR spectra of NOR and NOR@IONP$_{pH5}$ with focus on the 1000 to 2000 cm$^{-1}$ region Solid lines with values in red boxes highlight the FTIR spectral peaks for NOR while dashed lines indicate the corresponding wavenumbers in NOR@IONP$_{pH5}$.

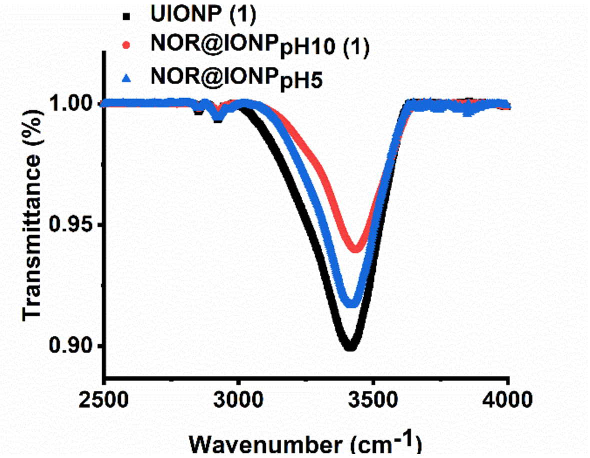


**Figure S2:** FTIR spectra in the range of 2500-4000 cm$^{-1}$ for UIONPs, NOR@IONP$_{pH10}$ and NOR@IONP$_{pH5}$.

## Modeling drug release for different samples

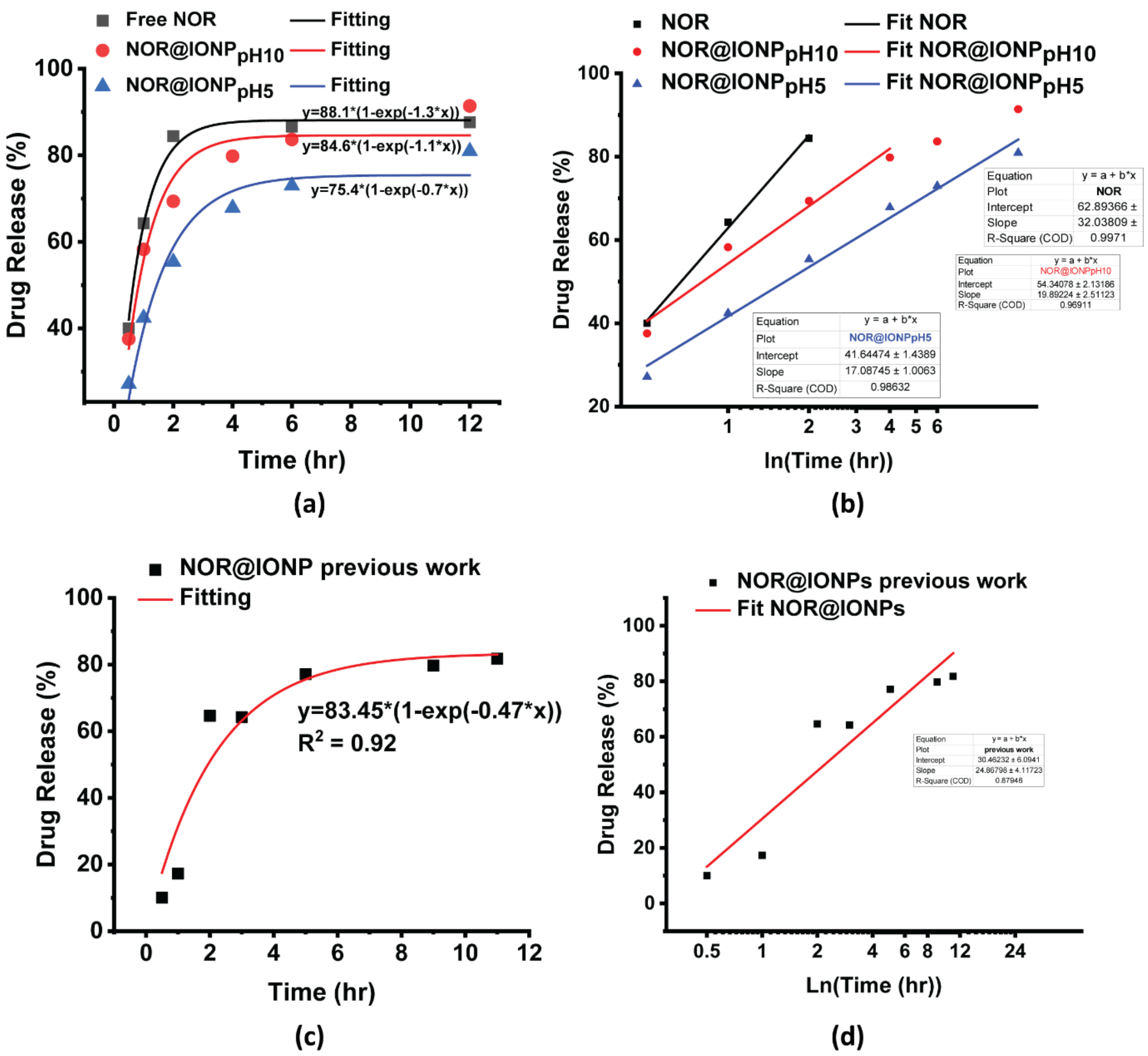


**Figure S3:** Drug release kinetic fitting using first order function or linear fitting for NOR and NOR@IONPs (a and b) from this work and (c and d) from previous work.

**Molecular structure of norfloxacin**

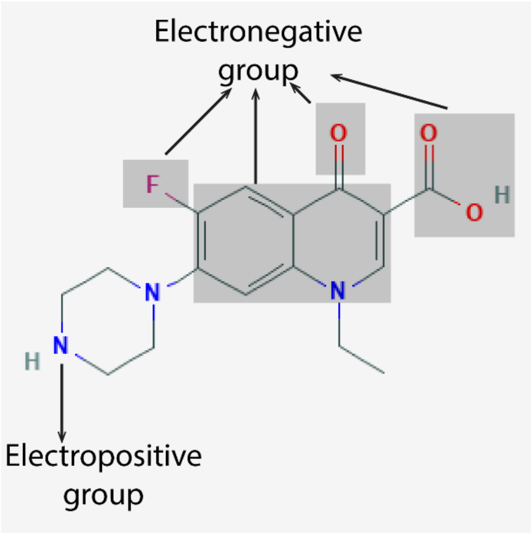


**Figure S4:** The structure of zwitterionic norfloxacin adapted from PubChem database (PubChem CID: 4539) indicating the electropositive and electronegative groups.

**Efficacy of NOR against *M. smegmatis* in RPMI media supplemented with 10% FBS (media used for macrophage culturing)**

0.5 $OD_{600}$ M. smegmatis culture was diluted (10 times) in RPMI media supplemented with 10% FBS. 200 µL of the diluted culture was taken in a 96 well plate and treated with 0 to 8 µg/mL of NOR for 48 h at 37 °C. Post treatment, 30 µL of 0.01% rezasurin was added to the culture and incubated for 8-10 h at 37 °C. Rezasurin is a blue dye that gets reduced in the presence of live cells to form a pink compound, resorfin. The inhibitory concentration of NOR is therefore identified to be 8 µg/mL, the concentration at which little or no change in rezasurin colouration occurred [Figure S2].

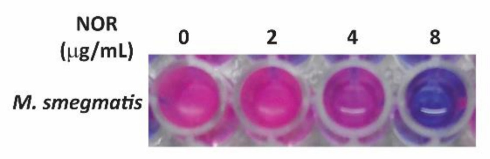


**Figure S5:** Rezasurin assay indicating growth (pink) and growth inhibition (purple) of *M. smegmatis* treated with NOR concentrations ranging from 0 to 8 μg/mL for 48 h.
